# Nutrient Digestibility, Chitin Recovery and Antioxidant Capacity of *Tenebrio molitor* Larvae Meal in Canine- and Feline-Adapted In Vitro Digestion Using a Conventional Fish Meal as References

**DOI:** 10.3390/ani16162462

**Published:** 2026-08-07

**Authors:** Matilda Rachele Dametti, Sara Frazzini, Irene Ferri, Matteo Dell’Anno, Cristina Spedale, Giancarlo Ruffo, Luciana Rossi

**Affiliations:** 1 Department of Veterinary Medicine and Animal Sciences, University of Milan, 26900 Lodi, Italy; matilda.dametti@unimi.it (M.R.D.); irene.ferri@unimi.it (I.F.); giancarlo.ruffo@unimi.it (G.R.); luciana.rossi@unimi.it (L.R.); 2 Department of Veterinary Sciences, University of Messina, Polo Universitario Annunziata, 98168 Messina, Italy; matteo.dellanno@unime.it (M.D.); cristina.spedale@studenti.unime.it (C.S.)

**Keywords:** *Tenebrio molitor*, companion animal nutrition, in vitro digestion, chitin, antioxidant activity, alternative proteins

## Abstract

The demand for sustainable protein sources for companion animal nutrition has stimulated interest in insect-derived ingredients. *Tenebrio molitor* larvae meal is a promising alternative to conventional proteins because of its nutritional composition and functional properties. This study evaluated the nutritional quality, in vitro digestibility, chitin digestibility, and antioxidant properties of *T. molitor* meal using canine- and feline-adapted in vitro digestion models. Chemical composition, amino acid and fatty acid profiles, total, protein, lipid, and chitin digestibility, antioxidant activity (ABTS), and Folin–Ciocalteu reducing capacity were determined. *T. molitor* meal contained 44.3% crude protein and 21.0% ether extract, with a balanced amino acid profile and a lipid fraction dominated by oleic and linoleic acids. Compared with the fish meal evaluated in this study, *T. molitor* showed higher total and lipid digestibility in both models, whereas protein digestibility was higher in dogs and comparable in cats. Chitin accounted for approximately 7.6% of the meal and showed negligible apparent digestibility. Water extracts of *T. molitor* exhibited greater antioxidant activity and reducing capacity than fish meal. These findings support the potential of *T. molitor* larvae meal as an alternative protein ingredient for companion animal nutrition, although in vivo studies are needed to confirm these observations.

## Simple Summary

The search for more sustainable protein sources has increased interest in insects as ingredients for pet food. Among these, the yellow mealworm (*Tenebrio molitor*) has attracted attention because of its nutritional characteristics, although information on its nutritional value for dogs and cats is still limited. In this study, we evaluated the nutritional composition of yellow mealworm meal and compared its digestibility, chitin content, and antioxidant properties with those of a commercial fish meal using laboratory models designed to simulate digestion in dogs and cats. Under the experimental conditions adopted, yellow mealworm meal showed higher overall and fat digestibility than the fish meal tested, while protein digestibility was higher in dogs and similar in cats. Chitin, the main structural component of the insect exoskeleton, showed little or no digestion, suggesting that it behaves as an indigestible dietary component. Yellow mealworm meal also exhibited higher antioxidant activity than fish meal before digestion, and its antioxidant properties were maintained after simulated digestion. Overall, these findings provide additional information on the nutritional characteristics of yellow mealworm meal and support its further evaluation as a protein ingredient for companion animal nutrition. Studies in live animals are still required to confirm these observations.

## 1. Introduction

In recent years, the global companion animal population has increased markedly, with approximately half of European households owning at least one pet in 2024, corresponding to more than 340 million animals [1,2,3]. This continuous growth has driven a substantial expansion of the pet food industry and, consequently, an increasing demand for high-quality protein ingredients with adequate nutritional value and sustainable supply chains suitable for companion animal nutrition. At the same time, concerns regarding the environmental impact and long-term sustainability of conventional livestock production have intensified the search for alternative protein sources capable of reducing the environmental footprint of pet food production while maintaining adequate nutritional quality [4,5].

Among the proposed alternatives, insects have emerged as promising feed ingredients because of their high feed conversion efficiency, limited land and water requirements, and comparatively low greenhouse gas emissions [6,7,8,9,10,11].

Within this group, *Tenebrio molitor* larvae have attracted particular attention owing to their favorable nutritional composition. Mealworm larvae are characterized by a relatively high protein content, a balanced indispensable amino acid profile, and a lipid fraction rich in oleic and linoleic acids, which may contribute not only to dietary energy supply but also to skin barrier function, coat condition and diet palatability [12,13,14].

Beyond their nutritional composition, insect-derived ingredients are increasingly being investigated as sources of bioactive compounds capable of exerting physiological effects beyond nutrient provision. In addition to proteins and lipids, *T. molitor* contains bioactive peptides, antioxidant compounds and chitin, a structural polysaccharide that has attracted considerable interest because of its potential interactions with intestinal physiology and immune function [13,14,15]. Consequently, research on insect meals has progressively expanded from the evaluation of their nutritional composition to the investigation of their functional properties in animal nutrition.

Among these bioactive components, chitin is one of the most distinctive constituents of insect-derived ingredients. Traditionally considered an anti-nutritional factor because of its limited digestibility in monogastric species, chitin is now recognized as a potential functional polysaccharide owing to its reported interactions with the intestinal microbiota, mucosal immunity and gastrointestinal physiology [15,16]. Nevertheless, its digestive fate under conditions representative of companion animals remains poorly characterized.

Likewise, although antioxidant activity has been reported for *T. molitor,* whether gastrointestinal digestion alters the bioaccessibility of antioxidant compounds remains largely unknown [17]. From a nutritional perspective, the value of an alternative protein ingredient depends not only on its chemical composition but also on the bioaccessibility of nutrients and the release, transformation or degradation of bioactive compounds during gastrointestinal digestion. Therefore, evaluating digestion is essential to estimate the actual nutritional quality and functional potential of insect-derived ingredients intended for companion animal nutrition.

Current knowledge on *T. molitor* is largely based on compositional analyses, whereas considerably less attention has been devoted to understanding how digestion affects nutrient availability and the release or persistence of functionally relevant compounds. This information is essential for assessing the actual nutritional value of insect-derived ingredients for companion animal nutrition. Moreover, most available studies have investigated these aspects separately, whereas integrated evaluations combining nutritional composition, species-adapted digestibility, chitin recovery, and antioxidant properties before and after digestion remain limited, particularly in the context of companion animal nutrition. Such an integrated approach may provide a more comprehensive assessment of the nutritional and functional potential of insect-derived ingredients.

Therefore, the present study aimed to comprehensively evaluate the nutritional and functional characteristics of *T. molitor* larvae meal in the context of companion animal nutrition. Specifically, chemical composition, amino acid and fatty acid profiles, nutrient digestibility using canine- and feline-adapted in vitro digestion models, apparent chitin digestibility, and antioxidant activity before and after digestion were investigated using a commercial fish meal as a conventional reference protein source.

## 2. Materials and Methods

### 2.1. T. molitor Larvae and Fish Meal Chemical Characterization

A representative sample of *T. molitor* larvae meal was obtained from a single production batch of larvae reared in a distributed insect-rearing prototype (UNIMI Prototype 01) installed at the Experimental Livestock Centre of the Department of Veterinary Medicine and Animal Sciences, Università degli Studi di Milano (Lodi, Italy). The prototype was developed in collaboration with Italian Cricket Farm within the EcoDigInsect research project, funded under the NODES—Nord Ovest Digitale e Sostenibile programme (Spoke 7). Larvae were reared on wheat bran under controlled environmental conditions (27 ± 2 °C; relative humidity 60–75%) for a 7-week production cycle. At the end of the rearing period, larvae were starved for 24 h and sieved to remove the residual substrate. They were subsequently euthanized by freezing at −20 °C and microwave-dried (CMG2071M, Candy Hoover Group, Brugherio, Italy) at a maximum input power of 120 W and a frequency of 2450 MHz for 5 min. Dried larvae were then ground using a laboratory mill to obtain the insect meal used for subsequent analyses. Before analysis, the meal was thoroughly mixed and homogenized, and subsamples were collected from different portions of the container to prepare a representative composite sample for all subsequent analyses. A commercial herring fish meal was selected as a conventional animal-derived protein ingredient for comparison (dry matter: 89.91%, as-is basis; crude protein: 74.73%, crude fat: 11.92%; ash: 16.79%, dry matter basis). A commercial herring fish meal was selected as the conventional animal-derived protein source for comparison because fish-based ingredients have historically represented a reference protein source in monoprotein and hypoallergenic pet foods and are characterized by high nutritional quality, a favorable fatty acid profile, and reported antioxidant properties [18,19].

Prior to the in vitro digestion assays, both meals were chemically characterized according to the Official Methods of Analysis of AOAC International [20]. Briefly, dry matter (DM) was determined by drying the samples in pre-weighed aluminum bags in a forced-air oven at 65 °C for 24 h, following AOAC method 930.15. Crude protein (CP) content was determined using the Kjeldahl method AOAC 2001.11. Protein content of *T. molitor* meal was calculated using the insect-specific nitrogen-to-protein conversion factor (Kp = 4.76), thereby minimizing overestimation due to the contribution of chitin-derived nitrogen [21]. Lipid content, expressed as ether extract (EE), was determined with ethyl ether in a Soxtec extractor according to AOAC method 2003.05. Total ash content was measured after incineration at 550 °C for 3 h, following AOAC method 942.05. Crude fiber (CF) was measured according to the AOCS Ba 6a-05 protocol. All analyses were performed in technical triplicates.

### 2.2. Aminoacid and Fatty Acid Profile Analyses of T. molitor Larvae Meal

A representative sample, prepared as described above, of the same batch of the *T. molitor* larvae meal was analyzed in technical triplicate to determine its aminoacid and fatty acid profiles. The aminoacid composition was determined by high-performance liquid chromatography (HPLC) following acid hydrolysis according to AOAC method 982.30. Sulfur-containing amino acids (methionine and cystine) were determined according to AOAC method 994.12, while tryptophan was determined after alkaline hydrolysis by HPLC with fluorescence detection (HPLC-FL) according to AOAC method 2017.03. Fatty acid composition was determined by capillary gas chromatography with flame ionization detection (GC-FID), according to AOAC method 996.06. All analyses were performed by an ISO/IEC 17025-accredited laboratory (Neotron SpA, Modena, Italy; Accredia certificate No. 0026).

### 2.3. Species-Adapted In Vitro Digestion Models

The standardized static INFOGEST in vitro digestion protocol, originally developed to simulate human gastrointestinal digestion, was adapted to approximate the gastrointestinal physicochemical conditions of dogs and cats [22]. Specifically, the composition of the simulated digestive fluids, including electrolyte and urea concentrations, as well as gastric pH and incubation temperature, was adjusted according to species-specific physiological characteristics, while preserving the static, sequential gastric–intestinal framework of the original INFOGEST protocol, following previously published species-specific adaptations [23]. The detailed composition of the canine- and feline-specific digestive fluids is reported below. Digestibility was subsequently assessed using the canine- and feline-adapted in vitro digestion models described below. Before digestion, all samples were dried at 65 °C until constant weight and ground to pass through a 1 mm sieve. For each ingredient and species-adapted digestion model, three independent in vitro digestion assays were performed, each using a separate incubation bottle as an experimental replicate.

For the canine model, 2 g of each sample were transferred into 250 mL glass bottles and mixed with 20 mL of ultrapure water. For the gastric phase, the pH was adjusted to 1.5 using 10 M HCl (1.00317, Merck Life Science, Darmstadt, Germany). Canine-specific simulated gastric fluid (SGF) was then prepared by adding 10 mL of a pepsin solution containing 2000 U/mL pepsin from porcine gastric mucosa (600–1800 U/mg; P7125, Sigma-Aldrich, St. Louis, MO, USA) and species-adapted electrolyte concentrations: 69.5 mM NaCl (1.06404, Merck Life Science, Darmstadt, Germany), 12.5 mM KCl (P3911, Sigma-Aldrich, St. Louis, MO, USA), 1 mM NaHCO_3_ (S6014, Sigma-Aldrich, St. Louis, MO, USA), 6 mM KH_2_PO_4_ (P0662, Sigma-Aldrich, St. Louis, MO, USA), 0.02 mM MgCl_2_ (M8266, Sigma-Aldrich, St. Louis, MO, USA), 1.9 mM CaCl_2_ (C5670, Sigma-Aldrich, St. Louis, MO, USA) and 15 mM urea (U5378, Sigma-Aldrich, St. Louis, MO, USA). Samples were incubated in a shaking water bath at 38.5 °C for 2 h. For the intestinal phase, the pH was adjusted to 7.5 using 1 M NaOH (1.06498, Merck Life Science, Darmstadt, Germany). Simulated intestinal fluid (SIF) was prepared by adding 10 mL of a trypsin-phosphate buffer solution (phosphate-buffered saline, pH 7.2–7.6; P4417, Sigma-Aldrich, St. Louis, MO, USA) containing 2500 U/mL trypsin from porcine pancreas (T4799, Sigma-Aldrich, St. Louis, MO, USA). Pancreatin (P3292, Sigma-Aldrich, St. Louis, MO, USA) and bile extract (B8631, Sigma-Aldrich, St. Louis, MO, USA) were subsequently added to obtain final concentrations of 50 and 30 mg/mL, respectively. Samples were further incubated under continuous shaking at 38.5 °C for 4 h. Enzymatic reactions were terminated by immersion of the incubation bottles in boiling water (100 °C). The undigested fraction was recovered by centrifugation at 3000× *g* for 10 min at 4 °C, washed twice with deionized water, centrifuged again at 3000× *g* for 5 min at 4 °C, and the resulting residue was dried at 65 °C until constant weight.

For the feline model, the same general procedure described for the canine-adapted model was applied, with species-specific modifications of gastric pH and simulated digestive fluid composition. For the gastric phase, 2 g of each sample were transferred into 250 mL glass bottles, mixed with 20 mL of ultrapure water, and the pH was adjusted to 2.0 using 10 M HCl (1.00317, Merck Life Science, Darmstadt, Germany). Feline-specific SGF was prepared by adding a pepsin solution containing 2000 U/mL pepsin from porcine gastric mucosa (P7125, Sigma-Aldrich, St. Louis, MO, USA) and species-adapted electrolyte concentrations: 34 mM NaCl (1.06404, Merck Life Science, Darmstadt, Germany), 13 mM KCl (P3911, Sigma-Aldrich, St. Louis, MO, USA), 1.4 mM NaHCO_3_ (S6014, Sigma-Aldrich, St. Louis, MO, USA), 1.8 mM KH_2_PO_4_ (P0662, Sigma-Aldrich, St. Louis, MO, USA), 0.04 mM MgCl_2_ (M8266, Sigma-Aldrich, St. Louis, MO, USA), 1.3 mM CaCl_2_ (C5670, Sigma-Aldrich, St. Louis, MO, USA) and 9.1 mM urea (U5378, Sigma-Aldrich, St. Louis, MO, USA). Samples were incubated in a shaking water bath at 38.5 °C for 2 h. For the intestinal phase, the pH was adjusted to 7.5 using 1 M NaOH (1.06498, Merck Life Science, Darmstadt, Germany), and 10 mL of the trypsin-phosphate buffer solution described for the canine model was added (phosphate-buffered saline, pH 7.2–7.6; P4417, Sigma-Aldrich, St. Louis, MO, USA; trypsin, T4799, Sigma-Aldrich). Pancreatin (P3292, Sigma-Aldrich, St. Louis, MO, USA) and bile extract (B8631, Sigma-Aldrich, St. Louis, MO, USA) were subsequently added to achieve final concentrations of 50 and 30 mg/mL, respectively. The electrolyte composition of the feline-specific SIF was adjusted to 30 mM NaCl (1.06404, Merck Life Science, Darmstadt, Germany), 11 mM KCl (P3911, Sigma-Aldrich, St. Louis, MO, USA), 31.2 mM NaHCO_3_ (S6014, Sigma-Aldrich, St. Louis, MO, USA), 0.1 mM MgCl_2_ (M8266, Sigma-Aldrich, St. Louis, MO, USA), 2.6 mM CaCl_2_ (C5670, Sigma-Aldrich, St. Louis, MO, USA) and 19.6 mM urea (U5378, Sigma-Aldrich, St. Louis, MO, USA). Samples were incubated under continuous shaking at 38.5 °C for 4 h.

Enzymatic activity was terminated, and the undigested fraction was recovered and dried as described for the canine-adapted model.

### 2.4. Post-Digestion Residue Characterization and Apparent Digestibility Calculations

To assess apparent in vitro digestibility, the undigested residues recovered after the canine- and feline-adapted digestion assays were dried at 65 °C until constant weight, weighed, and analyzed for chemical composition using the same analytical procedures described for the pre-digestion meal characterization. Apparent total in vitro digestibility (aTD):

(1) $$aTD\left(\%\right)=100-\left(\frac{final\;residue\;weight\times 100}{initial\;sample\;weight}\right),$$

Apparent in vitro digestibility of individual nutrients (aND) was calculated as:

(2) $$aND(\%)=100-\left(\frac{\%nutrient\;in\;final\;residue\times \left(100-TD\right)}{\%nutrient\;in\;initial\;sample}\right),$$

All nutrient concentrations used for digestibility calculations were expressed on a dry matter basis.

### 2.5. Chitin Determination in T. molitor Meal and In Vitro Digestion Residues

Chitin content in *T. molitor* larvae meal and in the undigested post-digestion residues recovered after the canine- and feline-adapted in vitro digestion assays was determined using the Acid Detergent Fiber minus Acid Detergent Lignin (ADF-ADL)-based procedure described by Hahn et al. [24]. This method was selected because it was applied in the analytical characterization of *T. molitor* larvae meal evaluated by the European Food Safety Authority (EFSA) in its Scientific Opinion on the safety of *Tenebrio molitor* larvae as a novel food [25]. Briefly, *T. molitor* larvae meal and post-digestion residues were dried at 65 °C until constant weight and subsequently ground using a laboratory mill to obtain a particle size < 1 mm. Prior to analysis, *T. molitor* larvae meal samples were defatted using n-hexane (1.04367, Merck Life Science, Darmstadt, Germany) as solvent (1:10, *w*/*v*) under continuous agitation for 30 min and then air-dried to remove residual solvent.

Approximately 1 g of each sample was transferred into pre-weighed filter crucibles (porosity 40 μm, 50 mL capacity). The crucibles were placed in a FIWE Raw Fiber Analyzer (Velp Scientifica, Usmate Velate, Italy), and 50 mL of ADF solution was added (Titolchimica S.p.a., Rovigo, Italy). Samples were boiled under reflux for 60 min. After digestion, the residue was resuspended in 50 mL of demineralized water preheated to 80 °C and allowed to soak for 5 min. The suspension was filtered under vacuum, and the washing step was repeated twice. An additional washing step with 50 mL of acetone (179124, Sigma-Aldrich, St. Louis, MO, USA) was then performed.

Following solvent removal, the crucibles containing the ADF residue were dried overnight and weighed. Subsequently, each crucible was transferred into a 150 mL glass beaker, and the filter cake was completely immersed in approximately 25 mL of 12 mol L^−1^ sulfuric acid (H_2_SO_4_) (84727, Merck Life Science, Darmstadt, Germany). The residue was gently homogenized using a glass stirring rod to ensure complete acid penetration. After 30 min, an additional 25 mL of sulfuric acid was added. The suspension was stirred intermittently and allowed to react for a total of 3 h. The mixture was then filtered under vacuum, and the residue was washed with hot demineralized water (80 °C) until neutral pH was reached, yielding the ADL residue.

The crucibles were dried overnight, weighed, and subsequently incinerated in a muffle furnace at 550 °C for 4.5 h. After cooling in a desiccator, the crucibles were weighed again. Chitin content was calculated as the ash-corrected difference between ADF and ADL residues and expressed on a dry matter basis.

Apparent in vitro chitin digestibility was calculated using the same apparent nutrient digestibility equation described above, based on the chitin content of the initial meal and of the undigested post-digestion residue.

### 2.6. Preparation of Aqueous Extracts and Digestion Supernatants

Aqueous extracts were prepared following the procedure described by Navarro Del Hierro et al. (2022), with minor modifications [25]. Briefly, *T. molitor* larvae meal and fish meal were mixed with ultrapure water at a 1:10 (*w*/*v*) ratio and incubating them at 70 °C for 30 min. The extracts and the supernatant fractions recovered after the in vitro digestion assays were centrifuged at 5000 rpm for 10 min and subsequently filtered through 0.45 μm membrane filters before antioxidant and Folin–Ciocalteu assays.

### 2.7. Antioxidant Activity Evaluation

The antioxidant activity of *T. molitor* larvae meal and fish meal was determined in aqueous extracts and post-digestion supernatants using the ABTS radical cation decolorization assay, as described by Re et al. [26]. The ABTS●^+^ (2,2′-Azino-bis(3-ethylbenzothiazoline-6-sulfonic acid) diammonium salt, A1888, Sigma-Aldrich, St. Louis, MO, USA; potassium persulfate, 216224, Sigma-Aldrich, St. Louis, MO, USA) working solution was diluted in deionized water to obtain an absorbance of 0.700 ± 0.02 OD at 734 nm at room temperature. Trolox ((±)-6-Hydroxy-2,5,7,8-tetramethylchromane-2-carboxylic acid, 238813, Sigma-Aldrich, St. Louis, MO, USA), a water-soluble analog of vitamin E, was used as the reference standard, and the calibration curve was generated by serial twofold dilutions ranging from 2000 μM to 0 μM.

For the assay, 10 μL of each sample (water extracts and supernatant fractions obtained after in vitro digestion) were added to 1 mL of the ABTS●^+^ working solution, and absorbance was recorded after 6 min of incubation in the dark. The evaluation was conducted by adding 10 μL of each prepared concentration to 1 mL of the ABTS●^+^ working solution. Absorbance readings were performed after 6 min of in the dark incubation. All measurements were carried out in triplicate.

All measurements were performed in technical triplicate. Antioxidant activity was expressed as percentage inhibition (PI%) according to the following equation:

(3) $$PI\left(\%\right)=\frac{\left({Abs}_{ABTS{●}^{+}}-{Abs}_{sample}\right)}{{Abs}_{ABTS{●}^{+}}}\times 100,$$

### 2.8. Folin–Ciocalteu Reducing Capacity

The Folin–Ciocalteu assay was used to estimate the total reducing capacity of aqueous extracts and post-digestion supernatants, conventionally reported as total phenolic content (TPC), according to Attard et al. [27]. Briefly, 10 μL of each sample were mixed with 100 μL of Folin–Ciocalteu reagent (F9252, Sigma-Aldrich, St. Louis, MO, USA) and 90 μL of sodium carbonate solution (1 M; 223530, Sigma-Aldrich, St. Louis, MO, USA). The reaction mixtures were incubated for 1 h at room temperature in the dark, and absorbance was measured at 765 nm using a microplate reader (Epoch, Agilent BioTek, Santa Clara, CA, USA).

Tannic acid (PHR3781, Merck Life Science, Darmstadt, Germany) was used as the calibration standard, and results were expressed as μg tannic acid equivalents (TAE) per g of sample.

### 2.9. Statistical Analysis

All statistical analyses were performed using GraphPad Prism version 10.3.1 (GraphPad Software, Boston, MA, USA). The experimental unit for digestibility and post-digestion analyses was the individual in vitro digestion bottle. Three independent digestion assays were performed for each ingredient within each species-adapted digestion model, whereas analytical determinations were carried out in technical triplicate as described above. Data normality was assessed using the Shapiro–Wilk test and homogeneity of variances was evaluated prior to parametric analyses.

Differences between *T. molitor* larvae meal and fish meal in apparent total, protein, and lipid in vitro digestibility were evaluated using unpaired *t*-tests within each species-adapted digestion model. Differences in antioxidant activity and Folin–Ciocalteu reducing capacity were evaluated using unpaired *t*-tests within each sample fraction, namely aqueous extracts, canine digestion supernatants, and feline digestion supernatants. Separate analyses were performed because the study was designed to compare the two protein sources within each digestion model rather than to evaluate the effects of species or ingredient × species interactions.

Results are expressed as mean ± standard deviation (SD), and differences were considered statistically significant at *p* < 0.05.

## 3. Results

### 3.1. Chemical Characterization of Meals

Proximate composition analysis of *T. molitor* meal showed a DM content of 96.84 ± 0.17%. CP, EE, ash, and CF contents on a DM basis were 44.33 ± 0.58%, 21.03 ± 2.0%, 4.82 ± 0.04%, and 12.79 ± 0.73%, respectively. The commercial herring fish meal used as reference ingredient showed a DM content of 95.28 ± 0.06%, with CP, EE, and ash contents of 71.05 ± 0.08%, 9.81 ± 0.08%, and 14.00 ± 0.27%, respectively, on a dry matter basis.

### 3.2. Amino Acid and Fatty Acid Profile Analyses of T. molitor Larvae Meal

The amino acid profile of *T. molitor* larvae meal showed the presence of the main indispensable amino acids relevant to canine and feline protein nutrition, together with several dispensable amino acids. Hydroxyproline was below the limit of quantification (Table 1).

The lipid fraction of *T. molitor* larvae meal was largely composed of unsaturated fatty acids, which accounted for approximately 77% of total fatty acids. Oleic acid (C18:1) and linoleic acid (C18:2) were the predominant fatty acids, representing 38.13 ± 0.47% and 34.27 ± 0.54% of total fatty acids, respectively. Saturated fatty acids represented approximately 23% of total fatty acids (Table 2).

### 3.3. Canine and Feline-Adapted In Vitro Digestibility

**Figure 1:**
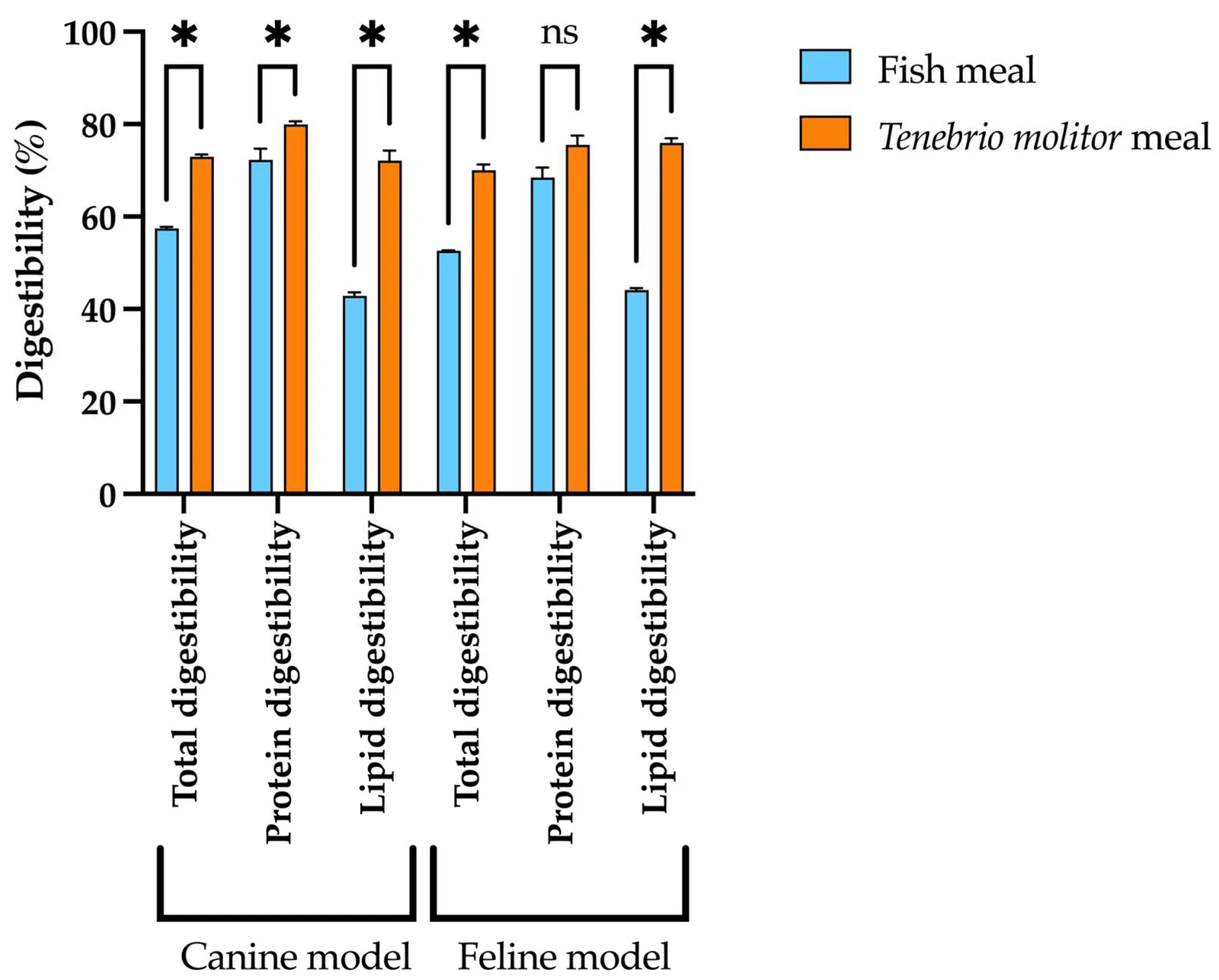
Apparent in vitro digestibility of *T. molitor* larvae meal and fish meal using the canine and feline-adapted digestion model. Bars represent mean values ± SD for total, protein, and lipid digestibility. Asterisks indicate statistically significant differences between ingredients (*p* < 0.05), whereas ns indicates no statistically significant difference (*p* ≥ 0.05).

In the canine-adapted digestion model, apparent total in vitro digestibility of *T. molitor* larvae meal was 72.97 ± 0.52%, significantly higher than that observed for the reference fish meal (57.18 ± 0.93%; *p* < 0.01). Apparent protein digestibility was also higher for *T. molitor* larvae meal than for fish meal (79.94 ± 0.72% vs. 72.30 ± 2.40%; *p* < 0.05). A similar pattern was observed for apparent lipid digestibility, with values of 72.10 ± 2.20% and 42.37 ± 1.34% for *T. molitor* larvae meal and fish meal, respectively (*p* < 0.01). Overall, under the experimental conditions adopted, *T. molitor* larvae meal showed higher apparent in vitro digestibility than the reference fish meal across all assessed fractions (Figure 1).

In the feline-adapted digestion model, apparent total in vitro digestibility of *T. molitor* larvae meal was 70.02 ± 1.31%, significantly higher than that of the reference fish meal (52.59 ± 0.14%; *p* < 0.01). Apparent lipid digestibility followed the same trend, with values of 75.93 ± 1.01% for *T. molitor* larvae meal and 44.11 ± 0.42% for fish meal (*p* < 0.01). Apparent protein digestibility was numerically higher for *T. molitor* larvae meal than for fish meal (75.57 ± 1.95% vs. 68.45 ± 2.15%), although this difference was not statistically significant. Within the experimental conditions tested, *T. molitor* larvae meal showed higher apparent in vitro digestibility than the reference fish meal for total matter and lipids in the feline-adapted model (Figure 1).

### 3.4. Chitin Content in T. molitor Meal and Apparent In Vitro Chitin Digestibility

The chitin content of *T. molitor* larvae meal was 7.58 ± 0.69% on an as-is basis. Following in vitro digestion, chitin accounted for 27.81 ± 1.55% of the undigested residue in the canine-adapted model and 25.69 ± 0.78% in the feline-adapted model. Apparent in vitro chitin digestibility was negligible, with values of 1.05 ± 0.57% and −0.01 ± 0.01% in the canine- and feline-adapted models, respectively.

### 3.5. Antioxidant Activity

**Figure 2:**
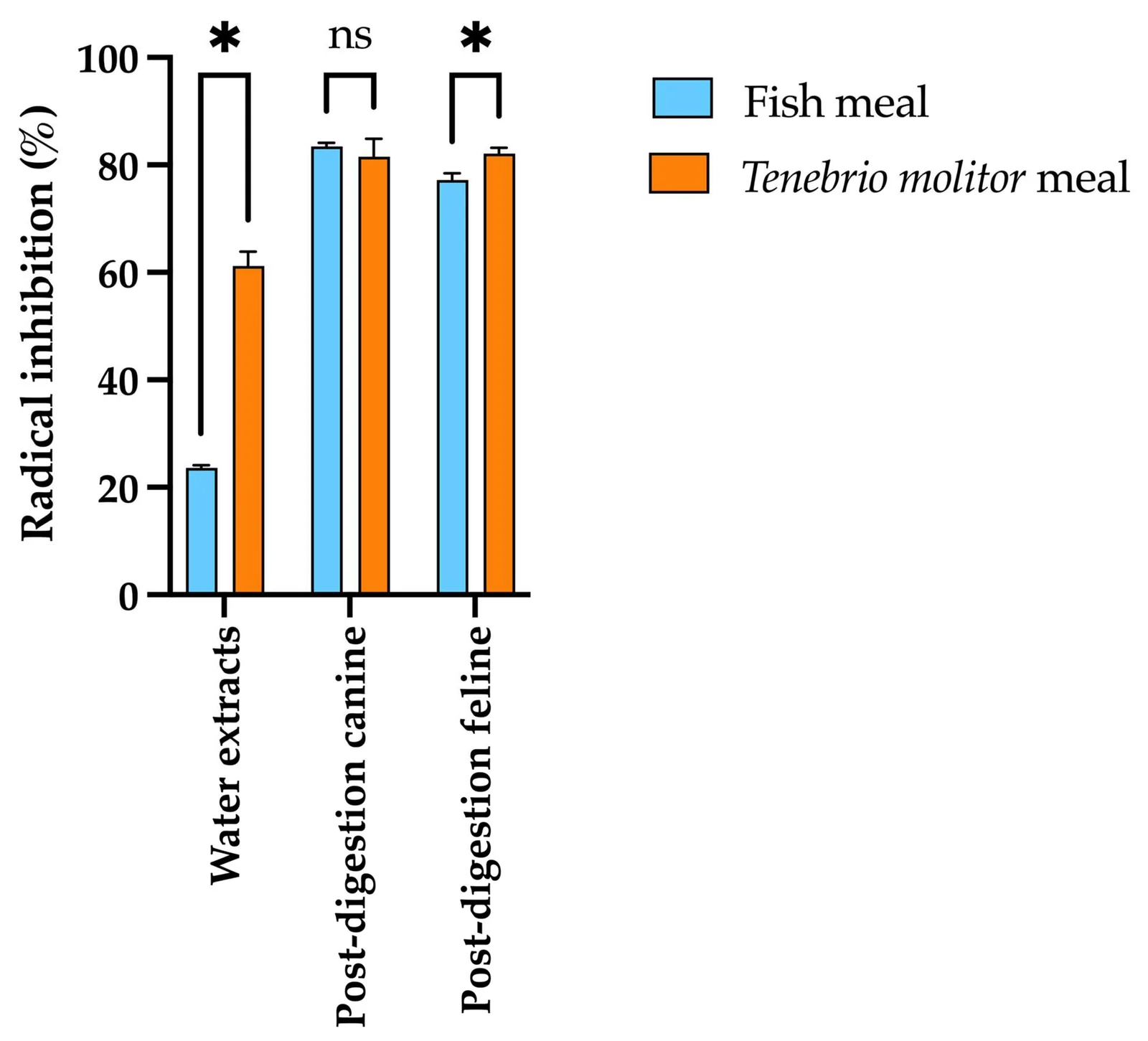
Antioxidant activity of *T. molitor* larvae meal and fish meal, expressed as percentage inhibition of the ABTS●^+^ radical. Bars represent mean values ± SD obtained from aqueous extracts and from post-digestion supernatants derived from canine- and feline-adapted in vitro digestion assays. Asterisks indicate statistically significant differences between ingredients (*p* < 0.05), whereas ns indicates no statistically significant difference (*p* ≥ 0.05).

Aqueous extracts of *T. molitor* larvae meal exhibited significantly higher antioxidant activity than fish meal, with ABTS●^+^ inhibition values of 61.17 ± 2.67% and 23.64 ± 0.49%, respectively (*p* < 0.01). Following in vitro digestion, antioxidant activity increased in both protein sources and in both species-adapted digestion models. In the canine-adapted model, post-digestion ABTS●^+^ inhibition values were comparable between *T. molitor* larvae meal and fish meal (81.57 ± 3.33% vs. 83.49 ± 0.66%). In the feline-adapted model, *T. molitor* larvae meal showed significantly higher antioxidant activity than fish meal (82.11 ± 1.13% vs. 77.17 ± 1.30%; *p* < 0.01). Overall, simulated gastrointestinal digestion increased the antioxidant activity of both meals, with *T. molitor* larvae meal maintaining high ABTS●^+^ radical inhibition in both species-adapted models (Figure 2).

### 3.6. Folin–Ciocalteu Reducing Capacity

**Figure 3:**
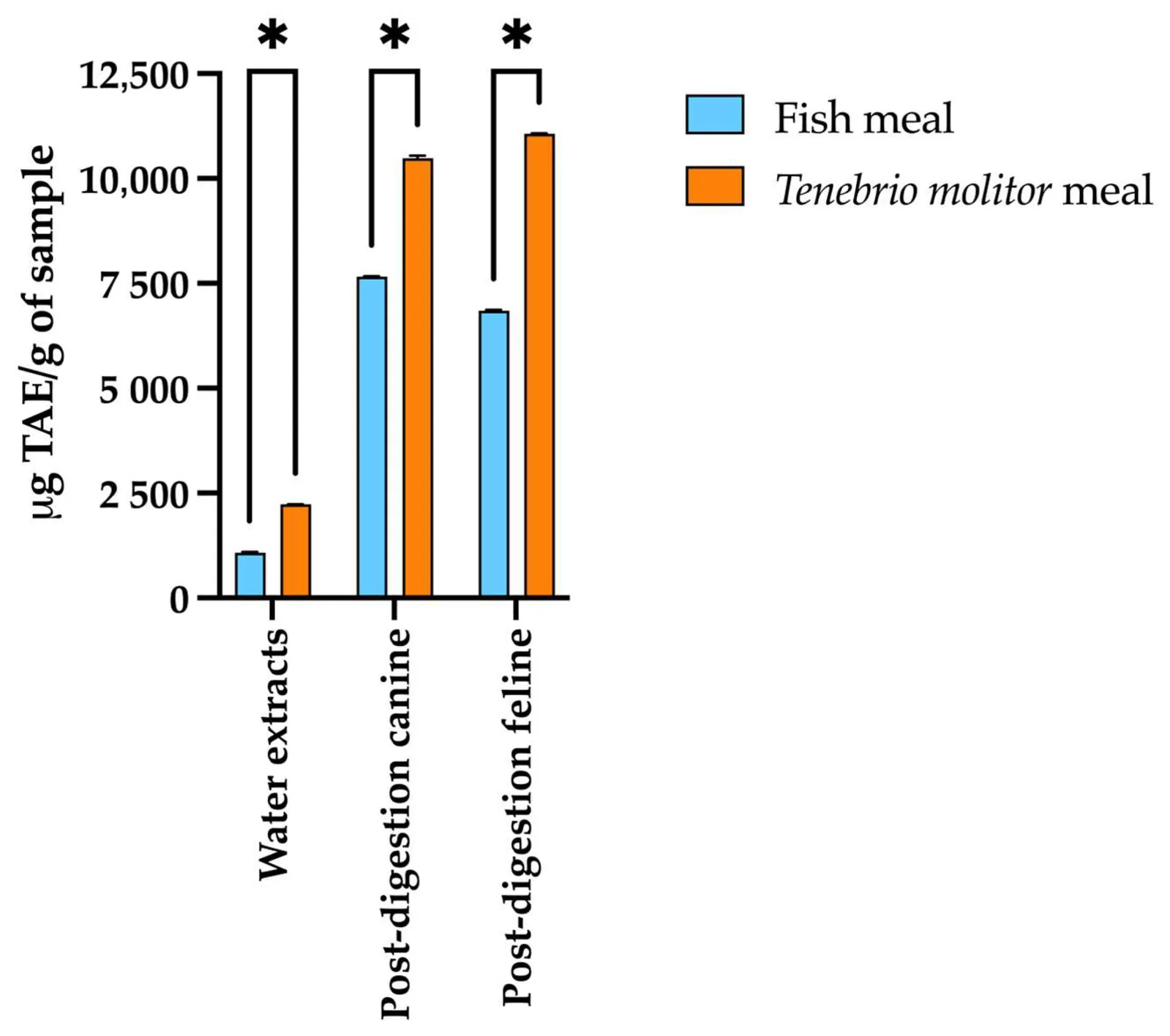
Folin–Ciocalteu reducing capacity of *T. molitor* larvae meal and fish meal, conventionally expressed as total phenolic content and reported as μg tannic acid equivalents (TAE) per g of sample. Bars represent mean values ± SD obtained from aqueous extracts and from post-digestion supernatants derived from canine- and feline-adapted in vitro digestion assays. Asterisks indicate statistically significant differences between ingredients (*p* < 0.05).

In aqueous extracts, *T. molitor* larvae meal showed significantly higher Folin–Ciocalteu reducing capacity, conventionally expressed as total phenolic content, than fish meal (2229.58 ± 1.30 vs. 1079.58 ± 15.36 μg TAE/g; *p* < 0.01). Following in vitro digestion, Folin–Ciocalteu reducing capacity increased markedly in both protein sources and in both species-adapted digestion models. In the canine post-digestion supernatants, values reached 10,486.77 ± 62.71 μg TAE/g for *T. molitor* larvae meal and 7663.33 ± 13.70 μg TAE/g for fish meal (*p* < 0.01). In the feline post-digestion supernatants, values were 11,063.33 ± 13.70 and 6550.83 ± 12.49 μg TAE/g for *T. molitor* larvae meal and fish meal, respectively (*p* < 0.01). Overall, *T. molitor* larvae meal showed higher Folin–Ciocalteu reducing capacity than fish meal across all experimental conditions (Figure 3).

## 4. Discussion

The present findings indicate that *T. molitor* larvae meal represents a nutritionally valuable ingredient for companion animal nutrition, exhibiting apparent in vitro nutrient digestibility values exceeding 70% together with antioxidant activity that was maintained or enhanced after simulated gastrointestinal digestion. While the study was conducted under in vitro conditions and therefore cannot predict physiological responses in vivo, the results suggest that gastrointestinal digestion does not impair the release or persistence of antioxidant compounds from *T. molitor* larvae meal. These observations support further investigation of this insect-derived ingredient as an alternative protein source for companion animals and provide additional information on its compositional and digestion-related characteristics beyond those currently available in the literature.

The proximate composition of *T. molitor* larvae meal obtained in the present study (DM 96.84%, CP 44.33%, EE 21.03%, ash 4.82% DM) was broadly consistent with values previously reported for yellow mealworm larvae meal, although some differences were observed [28,29]. Using the insect-specific nitrogen-to-protein conversion factor of 4.76, applied to reduce overestimation of protein content due to chitin-derived nitrogen, the crude protein concentration was comparable to that reported by Bosch et al. and slightly lower than that reported by Makkar et al. [28,29]. Accordingly, *T. molitor* larvae meal can be regarded as a moderate- to high-protein ingredient, whose practical nutritional value depends on inclusion level, diet formulation, processing conditions and amino acid bioavailability.

From a formulation perspective, the intermediate protein concentration of *T. molitor* larvae meal may offer flexibility in the development of complete diets, particularly when a single animal-derived protein source or a controlled protein contribution is required. However, such applications require evaluation at the complete-diet level, including amino acid balance, mineral profile, palatability and in vivo nutrient utilization.

A marked difference was observed in the lipid fraction compared with some previously published values. The EE content measured in the present study was lower than values reported by Bosch et al. and Makkar et al. [28,29]. This discrepancy may be partly explained by analytical methodology, because ether extract-based procedures generally recover mainly solvent-extractable lipids, whereas acid hydrolysis-based methods may also release bound lipid fractions. Differences in killing method, drying temperature, grinding intensity and post-harvest processing may also affect lipid extractability in insect meals [30]. Therefore, comparisons of lipid content among studies should be interpreted with caution unless similar analytical and processing conditions are used.

Nevertheless, *T. molitor* larvae meal contained a higher lipid fraction than the reference fish meal evaluated in this study. This characteristic may contribute to the energy density of diets containing *T. molitor.* Consequently, depending on the formulation objectives and the composition of the complete diet, the inclusion of this ingredient could potentially reduce the need for additional lipid sources. However, this hypothesis should be confirmed in complete diet formulation and feeding studies [31]. However, the nutritional implications of this lipid fraction depend not only on total fat content but also on fatty acid composition. In the present study, oleic acid and linoleic acid were the predominant fatty acids. This fatty acid profile is consistent with previous reports describing oleic and linoleic acids as the predominant fatty acids in *T. molitor* larvae meal, although their relative proportions may vary according to the rearing substrate, developmental stage and processing conditions [32,33]. Linoleic acid is particularly relevant in canine and feline nutrition because of its role in epidermal barrier function and skin integrity, whereas oleic acid contributes to the monounsaturated fatty acid fraction and may influence diet palatability and lipid quality [34,35,36]. Conversely, the absence or very low concentration of long-chain n-3 polyunsaturated fatty acids, such as EPA and DHA, represents a limitation compared with fish-derived ingredients. Therefore, when *T. molitor* meal is included also as lipid source in complete diets, complementary lipid sources such as fish oil, marine oils or algae-derived ingredients may be necessary to meet long-chain n-3 fatty acid requirements [37].

The ash content of *T. molitor* larvae meal was lower than that of the reference fish meal. This difference is consistent with the typically higher mineral content of fish meals, which may be influenced by the proportion of bone and mineralized tissues in the raw material [38]. A lower ash fraction may provide greater flexibility in mineral supplementation during diet formulation, especially when precise control of calcium, phosphorus and other minerals is required [39,40]. However, because the present study did not determine the detailed mineral profile or Ca:P ratio of *T. molitor* larvae meal, conclusions regarding mineral adequacy or suitability for specific clinical diets should be avoided.

The amino acid profile of *T. molitor* larvae meal showed the presence of the main indispensable amino acids relevant to canine and feline protein nutrition [41]. This supports its potential contribution to dietary protein quality, although the evaluation of amino acid composition alone is insufficient to establish protein adequacy in complete diets. Sulfur-containing amino acids, particularly methionine and cysteine, require careful consideration because they may become limiting depending on species, life stage, inclusion level and the amino acid contribution of the other ingredients. In the present study, methionine and cysteine/cystine concentrations were 0.809 and 0.639 g/100 g DM, respectively; therefore, their adequacy should be assessed at the complete-diet level rather than at the ingredient level alone.

The apparent in vitro digestibility results support the nutritional potential of *T. molitor* larvae meal under the experimental conditions adopted. In the canine-adapted model, *T. molitor* showed higher apparent total, protein and lipid digestibility than the reference fish meal. In the feline-adapted model, apparent total and lipid digestibility were significantly higher, whereas protein digestibility was numerically higher but not statistically different. These results indicate that the digestibility advantage of *T. molitor* was most consistent for total matter and lipids, while protein digestibility should be interpreted more cautiously, particularly in the feline model.

The higher apparent digestibility observed for *T. molitor* compared with the reference fish meal cannot be attributed to a specific factor based on the present data. However, previous studies indicate that several characteristics of commercial fish meals may influence their digestibility and therefore may have contributed to the differences observed in the present study. First, fish meals are highly heterogeneous ingredients, and their digestibility can vary depending on raw material quality, freshness, rendering conditions, drying temperature and ash content [42,43]. Excessive heat treatment may reduce protein accessibility through denaturation, aggregation or Maillard-type reactions, whereas high ash content may dilute the digestible organic fraction. Second, processing conditions applied to *T. molitor* larvae, including drying and milling, may have facilitated enzymatic access to nutrients [44]. Because these characteristics were not specifically evaluated for the commercial fish meal used in this study, these explanations should be regarded as plausible hypotheses based on previous literature rather than as demonstrated mechanisms. Therefore, the comparison reported here should be interpreted as a comparison between the specific *T. molitor* larvae meal and the specific commercial fish meal evaluated, rather than as a general demonstration that insect meal is intrinsically more digestible than fish meal.

These findings are consistent with previous studies reporting high digestibility and protein quality of *T. molitor* and other insect-derived ingredients [36,45,46]. However, the absolute digestibility values obtained in the present study were lower than those reported in some INFOGEST-based human digestion studies. This is not unexpected, because digestibility estimates are strongly affected by model design, including species-specific pH conditions, digestive fluid composition, enzyme activity, incubation time, matrix characteristics and the method used to recover and quantify the undigested fraction [22,47]. Therefore, while the absolute digestibility values reported here should not be directly compared with those obtained using the original human INFOGEST protocol, the relative differences observed between *T. molitor* larvae meal and the reference fish meal remain informative, as both ingredients were evaluated under identical canine- and feline-adapted experimental conditions. In this context, the present species-adapted models provide useful comparative information under canine- and feline-like physicochemical conditions, but they cannot be directly equated with in vivo digestibility or nutrient absorption.

Regarding chitin, the content observed in *T. molitor* larvae meal was consistent with the range previously reported for mealworm larvae, although chitin concentration may vary according to larval stage, rearing conditions, diet and analytical method [36]. In the present study, chitin accounted for approximately one quarter of the undigested residue after both canine- and feline-adapted digestion. Apparent in vitro chitin digestibility was negligible in both models, with values close to zero. This finding indicates that chitin was largely recovered in the insoluble residue after simulated gastric and small intestinal digestion.

This result is biologically plausible. Chitin is a β-1,4-linked polymer of N-acetyl-D-glucosamine, and its hydrolysis requires chitinolytic activity. Dogs and cats are carnivorous monogastric species and are expected to have limited capacity to degrade chitin during gastric and small intestinal digestion, particularly in the absence of microbial fermentation [48,49]. Accordingly, under the present experimental conditions, chitin should be regarded primarily as a poorly digestible structural polysaccharide rather than as a direct source of metabolizable energy or amino acids.

However, low digestibility should not be interpreted as lack of biological relevance. Chitin may behave similarly to an insoluble dietary fiber fraction and may influence digesta characteristics, microbial fermentation in the distal gut or host–microbiota interactions. Emerging evidence also suggests that chitin and chitin-derived compounds may interact with immune and intestinal pathways [50,51]. These potential effects could not be assessed using the present static gastric–intestinal in vitro approach, because the model did not include colonic fermentation, microbiota activity, epithelial absorption or immune endpoints. Therefore, the physiological relevance of chitin from *T. molitor* in companion animals remains an important topic for future in vivo and ex vivo studies.

The antioxidant assays performed in the present study provide chemical estimates of antioxidant capacity rather than direct evidence of biological antioxidant effects. Nevertheless, they represent one of the most relevant functional findings of the present study. Aqueous extracts of *T. molitor* larvae meal showed higher ABTS radical inhibition than fish meal before digestion. More importantly, simulated gastrointestinal digestion increased antioxidant activity in both protein sources, suggesting that digestion promoted the release or solubilization of compounds with radical-scavenging capacity. In the canine-adapted model, post-digestion antioxidant activity was comparable between *T. molitor* and fish meal, whereas in the feline-adapted model *T. molitor* maintained significantly higher ABTS inhibition.

Interestingly, the present study was not designed to identify the mechanisms underlying this species-specific pattern. However, differences in the physicochemical conditions of the two adapted digestion models, including gastric pH and digestive fluid composition, may have influenced the release or solubilization of antioxidant compounds during digestion [52,53]. Further studies are needed to clarify whether these differences reflect species-specific digestive processes or methodological aspects of the adapted in vitro models.

This increase after digestion may be explained by proteolysis and matrix disassembly. During simulated gastric and intestinal digestion, proteins and other macromolecular structures may be hydrolyzed, releasing peptides, amino acids and other low-molecular-weight compounds capable of donating electrons or hydrogen atoms in radical-scavenging assays [54,55]. Previous studies on *T. molitor* peptide extracts support the hypothesis that proteolytic treatment can enhance antioxidant activity by releasing bioactive peptides that are less accessible in the native matrix [56]. Therefore, the post-digestion increase in ABTS inhibition should be interpreted as an increase in antioxidant capacity of the soluble digesta fraction, rather than as direct evidence of antioxidant effects in vivo.

A similar interpretation applies to the Folin–Ciocalteu results. In the present study, *T. molitor* larvae meal showed higher Folin–Ciocalteu reducing capacity than fish meal both before and after digestion. However, the Folin–Ciocalteu assay is not specific for phenolic compounds and can react with several reducing substances, including peptides, amino acids, Maillard reaction products and other soluble reducing molecules [57,58]. Therefore, the post-digestion increase should be discussed as an increase in Folin–Ciocalteu reducing capacity, conventionally expressed as total phenolic content, rather than as a true increase in phenolic compounds. This distinction is particularly important in insect meals, where the phenolic fraction may derive partly from the rearing substrate or gut contents, while digestion may also release non-phenolic reducing compounds from the insect matrix.

The functional properties of insect meals are known to be influenced by multiple upstream and downstream factors. Rearing substrate composition, larval developmental stage, starvation period, killing method, drying conditions, milling and defatting can all modify nutrient composition, lipid oxidation, protein structure and the availability of antioxidant compounds. Therefore, the antioxidant and reducing capacity observed in the present study should be considered specific to the production batch, rearing substrate and processing conditions used. This point is particularly relevant for *T. molitor*, because substrate enrichment and post-harvest processing may be used to modulate the nutritional and functional profile of the final ingredient [59,60,61].

The present study has some limitations. First, only one batch of *T. molitor* larvae meal and one commercial fish meal were evaluated; therefore, ingredient variability could not be assessed. In addition, although the commercial fish meal was included as a conventional reference ingredient for the digestibility comparison, its amino acid and fatty acid profiles were not determined. Consequently, the comparison between the two protein sources should be interpreted primarily with respect to digestibility and functional properties rather than their complete compositional characteristics. Second, the static in vitro digestion models approximated selected canine and feline gastrointestinal conditions but did not reproduce absorption, metabolism, microbiota activity or host physiological responses. Finally, the study was conducted at the ingredient level and did not assess complete-diet digestibility, palatability, gastrointestinal tolerance or in vivo oxidative status. Overall, under the experimental conditions adopted, *T. molitor* larvae meal showed high apparent in vitro nutrient digestibility, negligible apparent chitin digestibility and antioxidant activity that was maintained or enhanced after simulated gastrointestinal digestion. These findings provide experimental support for the further evaluation of *T. molitor* larvae meal as an alternative insect-derived protein ingredient for companion animal nutrition. However, the observed functional properties should be considered hypothesis-generating rather than clinically demonstrated, and further in vivo studies are needed to determine whether the in vitro antioxidant responses, chitin recovery and nutrient digestibility observed here translate into measurable physiological benefits in dogs and cats.

## 5. Conclusions

In conclusion, under the experimental conditions adopted, *T. molitor* larvae meal showed high apparent in vitro nutrient digestibility in both canine- and feline-adapted digestion models. Compared with the reference fish meal evaluated in this study, *T. molitor* larvae meal exhibited higher apparent total and lipid digestibility in both models, while protein digestibility was higher in the canine model and comparable in the feline model. Chitin was largely recovered in the undigested residues and showed negligible apparent digestibility, indicating that it should be regarded as a poorly digestible structural component under gastric and small intestinal conditions rather than as a directly available nutrient. Simulated gastrointestinal digestion increased the antioxidant activity and Folin–Ciocalteu reducing capacity of the soluble fractions, suggesting the release or solubilization of compounds with reducing and radical-scavenging activity during digestion. Overall, these findings provide experimental support for the further evaluation of *T. molitor* larvae meal as an alternative animal-derived protein ingredient for companion animal nutrition. However, in vivo studies are required to confirm nutrient utilization, palatability, gastrointestinal tolerance and the biological relevance of the functional responses observed in vitro.

## Figures and Tables

**Table 1:** Amino acid composition of *T. molitor* larvae meal. Values are expressed as g/100 g dry matter (mean ± SD).

Amino Acid	Content (g/100 g of Sample DM)
Alanine	4.55 ± 0.73
Arginine *	3.47 ± 0.56
Aspartic acid, including asparagine	4.92 ± 0.78
Cysteine and cystine, expressed as cysteine *	0.639 ± 0.102
Glutamic acid, including glutamine	6.95 ± 1.12
Glycine	3.25 ± 0.52
Histidine *	1.75 ± 0.28
Isoleucine *	2.69 ± 0.43
Leucine *	4.57 ± 0.73
Lysine *	3.46 ± 0.56
Methionine *	0.809 ± 0.129
Phenylalanine *	2.11 ± 0.34
Proline	4.55 ± 0.73
Serine	2.92 ± 0.46
Threonine *	2.54 ± 0.40
Tryptophan *	0.742 ± 0.119
Tyrosine	3.96 ± 0.63
Valine *	3.83 ± 0.61

* Essential amino acids relevant to canine and feline protein nutrition.

**Table 2:** Fatty acid composition of *T. molitor* larvae meal. Values are expressed as percentage of total fatty acids (mean ± SD). Arachidonic, butyric, capric, caproic, caprylic, decenoic, dihomo-gamma-linolenic, docosadienoic, docosahexaenoic, docosapentaenoic, docosatetraenoic, dodecenoic, eicosapentaenoic, eicosatrienoic, erucic, heneicosanoic, heptanoic, lignoceric, methyl hexadecanoic, methyl pentadecanoic, methyl tetradecanoic, methyl tridecanoic, nervonic, pentadecanoic, stearidonic, tridecanoic resulted below the limit of quantification.

Fatty Acid	Content (% of Total Fatty Acids)
**Saturated fatty acids (SFA)**	22.61 ± 0.44
Arachidic acid (C20:0)	0.34 ± 0.03
Behenic acid (C22:0)	0.15 ± 0.02
Heptadecanoic acid (C17:0)	0.25 ± 0.02
Lauric acid (C12:0)	0.18 ± 0.02
Myristic acid (C14:0)	2.18 ± 0.16
Palmitic acid (C16:0)	16.04 ± 0.40
Stearic acid (C18:0)	3.47 ± 0.12
**Monounsaturated fatty acids (MUFA)**	40.96 ± 0.49
11-eicosenoic acid (C20:1)	0.16 ± 0.02
Heptadecenoic acid (C17:1)	0.17 ± 0.02
Myristoleic acid (C14:1)	0.17 ± 0.02
Oleic acid (C18:1, including geometric and positional isomers)	38.13 ± 0.47
Palmitoleic acid (C16:1, including geometric and positional isomers)	2.33 ± 0.13
**Polyunsaturated fatty acids (PUFA)**	36.43 ± 0.56
11,14-eicosadienoic acid (C20:2)	0.10 ± 0.01
α-Linolenic acid (C18:3, including geometric and positional isomers)	2.06 ± 0.15
Linoleic acid (C18:2, including geometric and positional isomers)	34.27 ± 0.54
**Total n-3 fatty acids**	2.06 ± 0.15
**Total n-6 fatty acids**	34.37 ± 0.54
**n-6/n-3 ratio**	16.68

## Data Availability

All data are available within the manuscript and from the corresponding author upon reasonable request.

## Abbreviations

The following abbreviations are used in this manuscript:

*T. molitor*	*Tenebrio molitor*
ABTS	2,2′-azino-bis(3-ethylbenzothiazoline-6-sulfonic acid
AOAC	Association of Official Analytical Chemists
CP	Crude Protein
EE	Ether Extract
CF	Crude Fiber
AOCS	American Oil Chemists’ Society
HPLC	High-Performance Liquid Chromatography
GC	Gas Chromatography
SGF	Simulated Gastric Fluid
SIF	Simulated Intestinal Fluid
aTD	apparent Total Digestibility
aND	apparent Nutrient Digestibility
ADF	Acid Detergent Fiber
ADL	Acid Detergent Lignin
PI	Percentage Inhibition
TAE	Tannic Acid Equivalents
DM	Dry Matter
EPA	Eicosapentaenoic acid
DHA	Docosahexaenoic acid
